# Gut Microbiome in Down Syndrome

**DOI:** 10.1371/journal.pone.0112023

**Published:** 2014-11-11

**Authors:** Elena Biagi, Marco Candela, Manuela Centanni, Clarissa Consolandi, Simone Rampelli, Silvia Turroni, Marco Severgnini, Clelia Peano, Alessandro Ghezzo, Maria Scurti, Stefano Salvioli, Claudio Franceschi, Patrizia Brigidi

**Affiliations:** 1 Department of Pharmacy and Biotechnology, University of Bologna, Bologna, Italy; 2 Institute of Biomedical Technologies - Italian National Research Council, Milan, Italy; 3 Department of Experimental, Diagnostic and Specialty Medicine, University of Bologna, Bologna, Italy; 4 Interdepartmental Centre “L. Galvani” (CIG), University of Bologna, Bologna, Italy; University of Palermo, Italy

## Abstract

**Background:**

Premature aging seriously compromises the health status of Down Syndrome (DS) persons. Since human aging has been associated with a deterioration of the gut microbiota (GM)-host mutualism, here we investigated the composition of GM in DS.

**Methods:**

The observational study presented involved 17 adult DS persons. We characterized the GM structure by 454 pyrosequencing of the V4 region of the 16S rRNA gene. DS microbiome was compared with that of age-matched healthy non-trisomic adults enrolled in the same geographic area.

**Results and Conclusions:**

The dominant GM fraction of DS persons showed an overall mutualistic immune-modulatory layout, comparable to that of healthy controls. This makes GM a possible factor counteracting the genetic determined acceleration of immune senescence in DS persons. However, we also found detectable signatures specific for DS among subdominant GM components, such as the increase of *Parasporobacterium* and *Sutterella*. In particular, the abundance of this last microorganism significantly correlated with the Aberrant Behavior Checklist (ABC) total score, allowing us to hypothesize a possible role for this microbial genus in behavioral features in DS.

## Introduction

Resulting from the trisomy of the 21^st^ chromosome, Down Syndrome (DS) represents the most common genetic cause of intellectual disability. The incidence of DS is of 14.47 per 10,000 live births, with higher incidence for increasing maternal age [1]. Even if lifespan of persons with DS is significantly increased in the past decades - from 12 years of age in the 1940s to a mean life expectancy of 60 years today – DS persons exhibit sign of premature aging [2]. Thus, the overall increase in life expectancy in DS comes with a parallel increase of risk for age-related diseases, such as Alzheimer’s disease, increased rate of infections, hypertension and obesity [3], [4]. This results in a considerable need of medical, rehabilitative and social services, as well as a compromised health status and premature death.

The acceleration of biological aging in DS principally involves the increase in the oxidative stress [5], as well as a premature immunosenescence in the immune system [6]. In particular, adult DS persons show typical signs of immunosenescence which are normally found in much older non-trisomic individuals, including alterations of B and T cell subpopulations [7], [8], and they have higher local inflammation with respect to age-matched non-trisomic subjects [9], [10]; however it is not clear whether DS is characterized by the age-related increase of pro-inflammatory status - known as inflamm-aging - found in the elderly [11]–[13].

Human aging and immunosenescence are accompanied by a deterioration of the mutualistic relationship with the gut microbiota (GM) [14]–[16]. Encompassing the reduction of key immunomodulating groups as members of the butyrate producers *Clostridium* clusters IV and XIVa [17], and the concomitant increase of pro-inflammatory pathobionts [18], age-dependent GM dysbioses result in an overall pro-inflammatory configuration of the ecosystem capable to boost a self-sustained pro-inflammatory loop favoring immunosenescence [14]. In this context, we investigated - to our knowledge for the first time - the GM structure in DS persons, under the hypothesis that the GM structure of these subjects could present alterations typical of an aged-type microbiota which favors immunosenescence.

Autism Spectrum Disorder (ASD) features are also quite common in DS persons, among which behavioral problems are prominent [19]. In particular, higher total score of the Aberrant Behavior Checklist (ABC), a rating scale specifically designed for individuals with severe intellectual disability [20], occurs in DS persons with ASD [21]. Since in recent years an association between ASD and specific GM genera has been outlined [22], [23], the characterization of the GM ecosystem in DS persons allows us to further explore the association between GM and ASD.

Here we characterized the GM structure in 17 adult DS persons by 454 pyrosequencing of the V4 region of the 16S rRNA gene. In order to highlight deviations from a healthy-like GM structure, the gut microbial ecosystem of DS was compared with the previously published GM profiles of 16 age-matching healthy adults [24]. Furthermore, in order to look for any alterations of the DS microbiota ecosystem towards an aged-type configuration, a GM meta-analysis also including elderly and centenarians from Rampelli *et al.* [25] was performed. According to our data, the dominant GM fraction of DS persons showed an overall immune-modulatory layout, comparable to that of healthy adults. However, signatures specific for DS gut microbiome were detectable among the subdominant GM components and associated with the ABC total score. This suggests that such components are correlated to behavioral features in DS as already demonstrated in subjects with diagnosed ASD.

## Materials and Methods

### Subject enrolment and sample collection

Persons with DS were enrolled prospectively from 2008, in the framework of an open-label study on DS. The study was approved by the local Ethics Committee (S. Orsola Hospital, University of Bologna; ethical clearance #126/2007/U/Tess, released on December 18, 2007). A written informed consent to participate to the study was obtained from DS persons and from their parents or relatives (brothers/sisters). DS persons were recruited with the help of CEPS, OPIMM and ANFFAS, three local non-profit associations dealing with DS persons operating in the eastern part of Emilia-Romagna Region (Bologna and Ferrara provinces). Participation to the study was totally on a voluntary basis, with no reward for the participants or their families. A standard questionnaire to collect information regarding the health status, drugs use, clinical anamnesis, and lifestyle was administered. In order to take account of the dietary habits of DS persons, 24-hour dietary recalls were provided for each of the enrolled subjects for 3 days. Subjects affected by malignant neoplasia and/or in therapy with immunosuppressive drugs like cyclosporin, methotrexate, glucorticoids, anticoagulant drugs, and who recently (at one month) used antibiotics and/or probiotics were excluded from the study. Stool samples from 17 adult DS persons randomly selected from this group were used for this study. Feces were collected and stored at −80°C and analyses were performed within 3 months.

### Neuropsychiatric assessment and adaptive behavior assessment

The cognitive level of 13 DS persons was evaluated with Wechsler Scale and resulted to be within the range of Cognitive Impairment (IQ scores below 70), as expected. For the remaining 4 DS persons, the cognitive level was assessed by using the mental age values extrapolated from VABS (see below). DS persons were classified within 4 levels of Cognitive Impairment (mild, moderate, severe and profound) according to DSM IV criteria.

For neuropsychiatric assessment, the ABC (Aberrant Behavior Checklist) [20] test was used. The italian version of ABC was used during verbal interview to care givers (parents and/or siblings, or educational personnel in one case). ABC is a 58-item caregiver report checklist that assesses maladaptive behaviors in individuals with developmental disabilities using a simple four-point rating scale (0–3) with higher scores reflecting more problems. ABC items are grouped into five subscales: (1) Irritability, Agitation, Crying, (2) Lethargy, social withdrawal, (3) Stereotypic behavior, (4) Hyperactivity, non-compliance, and (5) Inappropriate speech.

For adaptive behavior assessment, the Vineland Adaptive Behavior Scales (VABS) [26], [27] were used. The test assesses personal autonomy and social responsibility of individuals from birth to adulthood through questions designed to assess the skills actually possessed by the subject at the time of evaluation. The scales are used with both non-disabled and disabled persons. The questionnaire in its complete form consists of 540 items that include the following areas: communication, daily living skills, socialization, motor skills. The questionnaire was directly administered to parents, or whoever takes care of the DS person.

### Microbial DNA extraction from feces

Total bacterial DNA from fecal material was extracted using QIAamp DNA Stool Mini Kit (QIAGEN) with a modified protocol [28]. Briefly, 250 milligrams of feces were suspended in 1 mL of lysis buffer (500 mM NaCl, 50 mM Tris-HCl pH 8, 50 mM EDTA, 4% SDS). Four 3-mm glass beads and 0.5 g of 0.1-mm zirconia beads (BioSpec Products) were added, and the samples were treated in FastPrep (MP Biomedicals) at 5.5 movements per second for 1 min for 3 times, with 5-min intervals in ice. Samples were heated at 95°C for 15 min, then centrifuged for 5 min at full speed to pellet stool particles. Supernatants were collected and 260 µL of 10 M ammonium acetate was added, incubated in ice for 5 min and centrifuged at full speed for 10 min. One volume of isopropanol was added to each sample and incubated in ice for 30 min. The precipitated nucleic acids were collected by centrifugation for 15 min at full speed and washed with 70% ethanol. Pellets were resuspended in 100 µL of TE buffer and treated with 2 µL of DNase-free RNase (10 mg/mL) at 37°C for 15 min. Proteins were removed by proteinase K treatment, and DNA was subsequently purified following the manufacturer’s instructions. Final DNA concentration was measured by using NanoDrop ND-1000 (NanoDrop Technologies).

### 16S rRNA gene amplification

For the amplification of the V4 region of the 16S rRNA gene the primer set 520F (5′-AYTGGGYDTAAAGNG-3′) and 802R (5′-TACNVGGGTATCTAATCC-3′) (with Y = C/T, D = A/G/T, N = any base, V = A/C/G) [29] was utilized. Primers included at their 5′ end the adaptor sequence used in the 454-sequencing library preparation protocol, linked to a unique MID tag barcode of 10 bases that allowed the identification of the different samples. PCR mixtures contained 0.5 µM of each forward and reverse primer, 100 ng of template DNA, 2.5 U of GoTaq Flexi Polymerase (Promega), 200 µM of dNTPs and 2 mM of MgCl_2_ in a final volume of 50 µL. Samples were initially denatured at 95°C for 5 min, followed by 35 cycles of 94°C for 50 s, 40°C for 30 s, and 72°C for 60 s, with a final extension step at 72°C for 5 min [24]. PCR amplifications were carried out in a Biometra Thermal Cycler T Gradient (Biometra).

### Pyrosequencing

16S rRNA gene V4 hypervariable region amplicons were purified using MinElute PCR Purification Kit (QIAGEN) and quantified using the Quant-iT PicoGreen dsDNA Kit (Invitrogen). Amplicons were pooled in equal amounts (creating one 9-plex and one 8-plex pools) and again purified by 454-Roche Double Ampure size selection protocol with Agencourt AMPure XP DNA purification beads (Beckman Coulter Genomics GmbH) to remove primer dimers, according to the manufacturer’s instructions (454 LifeSciences, Roche). Before emulsion PCR, the purified amplicon pools were quantified using a quantitative Real Time PCR (qPCR) by KAPA Library Quant Kits (KAPA Biosystems). Afterwards pools were fixed to microbeads to be clonally amplified by emulsion PCR following the GS-FLX protocol Titanium emPCR LIB-A (454 LifeSciences, Roche). Beads were enriched in order to keep only those carrying identical PCR products on their surface, and loaded onto a picotiter plate for pyrosequencing reactions, following the GS-FLX Titanium sequencing protocol. Each pool was sequenced in one eighth of a plate. Amplicon sequences were deposited in MG-RAST under the project ID 10557 (http://metagenomics.anl.gov/linkin.cgi?project=10557).

### Bioinformatic analysis and statistical data analysis

Sequencing reads were analyzed using the QIIME pipeline as described previously [24]. Briefly, V4 sequences were filtered according to the following criteria: (i) read length not shorter than 150 bp and not longer than 350 bp; (ii) no ambiguous bases (Ns); (iii) a minimum average quality score over a 50-bp rolling window of 25; (iv) exact match to primer sequences and maximum 1 error in barcode tags. RDP-classifier (version 2.2) with 50% as confidence value threshold was used for bacterial taxonomy assignment. Trimmed reads were clustered into OTUs at 97% identity level and further filtered for chimeric sequences using ChimeraSlayer (http://microbiomeutil.sourceforge.net/#A_CS). Alpha-diversity and rarefaction plots were computed using Shannon, PD whole tree, Chao1 and observed species metrics. Weighted and unweighted UniFrac distances were used to perform Principal Coordinates Analysis (PCoA). PCoA and heatmap were built using R packages Made4 [30], and Vegan (http://cran.r-project.org/package=vegan).

The R packages Stats and Vegan were used to perform statistical analysis. In particular, to compare the GM structure between DS persons and healthy adults, Wilcoxon signed rank test was used. Data separation in the PCoA was tested using a permutation test with pseudo F-ratios (function Adonis in the Vegan package). Significant differences in phylum- or genus-level abundance between DS persons and healthy controls were assessed by Mann-Whitney U tests, and corrected for multiple comparisons using the Benjamini-Hochberg method when appropriate. PCoA of the Euclidean distances of GM genera between DS persons, 16 healthy Italian adults from Schnorr *et al.* [24], and 5 elderly and 3 centenarians from Rampelli *et al.* [25] was carried out by using Vegan. The Kendall correlation test between ABC score and the relative abundance of genera in DS microbiome was achieved using function cor.test of the R package Stats. In all statistical tests, *P*<0.05 was considered as statistically significant.

## Results

### Intestinal microbiota in DS persons

**Figure 1:**
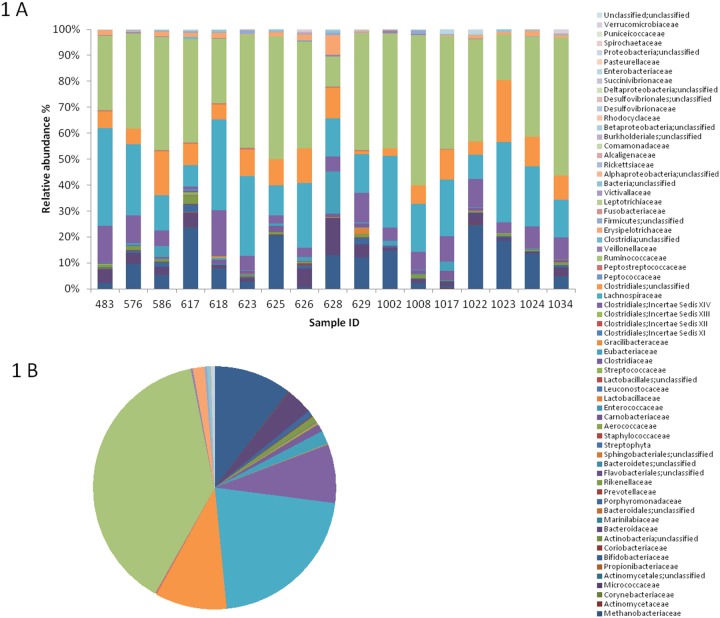
Individual (A) and average (B) gut microbiota profiles of the enrolled DS persons. Relative abundance of family-level assigned OTUs is reported. Colors were assigned for all families detected.

Seventeen adult DS persons (19–35 years, 7 males and 10 females) randomly selected from a larger cohort participated to this study. The main characteristics of these subjects are provided in Table S1. As expected, they were in general overweight (mean BMI 27.05), while problems in alvus or eating were reported for 7 out of 17 subjects (41%). Stools were collected and GM structure was characterized by means of pyrosequencing of the V4 region of the bacterial 16S rRNA gene (Figure 1A). A total of 67,029 high-quality reads were obtained with a mean of 3,943 reads per subject. Reads were clustered in 7,457 operational taxonomic units at 97% of identity. OTU rarefaction curves based on number of unique OTUs were obtained with different phylogenetic diversity metrics, such as Shannon, Chao1, PD whole tree and observed species; curves reached the plateau, approximating the saturation level, after 2,400 reads (see Figure S1).

According to our data, the GM of DS persons was largely dominated by Firmicutes (relative abundance (rel. ab.) 83%*±*2), Actinobacteria (rel. ab. 9%*±*1.6) and Bacteroidetes (rel. ab. 8%*±*1.8). With rel. ab. values below 5%, Proteobacteria, Verrucomicrobia and Fusobacteria were largely subdominant phyla. The most represented families in DS gut microbial communities were *Ruminococcaceae* (rel. ab. 39%±3), *Lachnospiraceae* (rel. ab. 20%±1.4), *Clostridiales* (rel. ab. 9%±1), *Clostridiales Incertae Sedis XIV* (rel. ab. 8%±1.2), *Bifidobacteriaceae* (rel. ab. 8%±1.5) and *Bacteroidaceae* (rel. ab. 5%±1.4) (Figure 1B). Subdominant families showing rel. ab. values below 5% were *Eubacterium*, *Porphyromonadaceae*, *Rikenellaceae*, *Erysipelotrichaceae*, *Veillonellaceae*, *Alcaligenaceae*, and *Verrucomicrobiaceae*.

No separation of the DS microbiota structure according to BMI [31] was found (*P*>0.05) (see Figure S2).

### Comparison between GM communities in DS persons and healthy non-trisomic adults

In order to highlight possible GM signatures of DS, the microbiota structure from the 17 DS persons enrolled in the present study was compared with that previously published from 16 healthy young adults aged between 20 and 40 years [24]. To exclude any country- and age-related effect on microbiota structure, as well as biases due to a study effect [32], age-matched Italian adults were selected for comparison. Both DS persons (see Table S2) and healthy adults [24] adhered to the standard Mediterranean diet, abundant in plant foods, fruit, pasta, bread and olive oil, and low-to-moderate in dairy and red meat.

**Figure 2:**
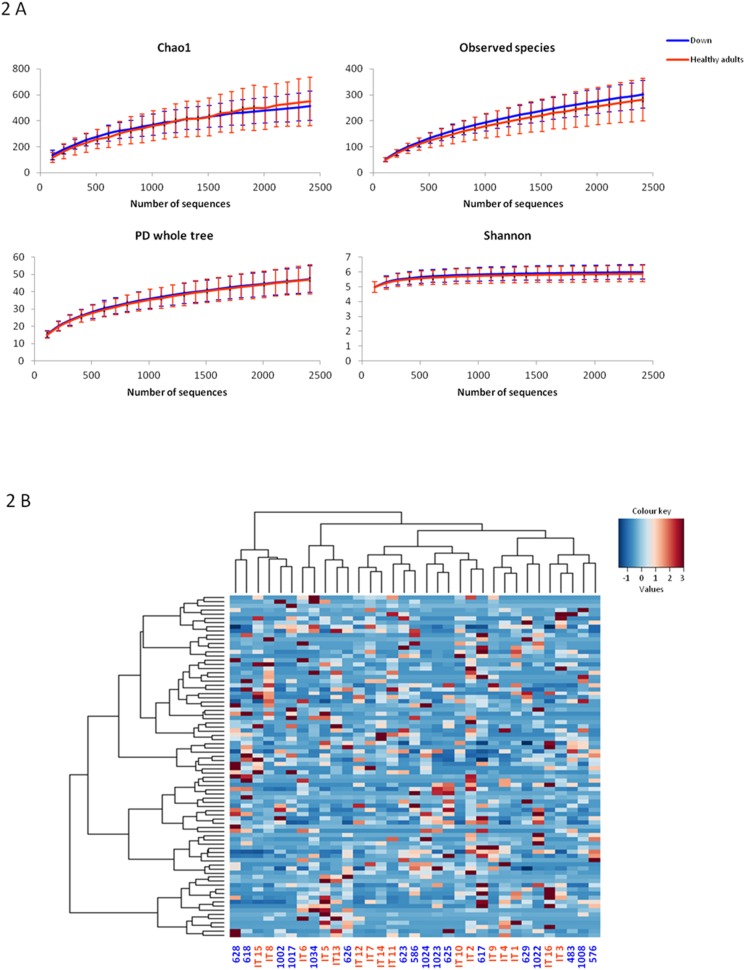
Similarity between the gut microbiota of DS persons and healthy non-trisomic adults for diversity and overall community composition. (A) Superimposition of the rarefaction curves of different α-diversity metrics (Faith’s phylogenetic diversity (PD whole tree), observed OTUs, the Chao1 measure of microbial richness, and the Shannon index of biodiversity). (B) Hierarchical Ward-linkage clustering based on the Spearman correlation coefficients of genus proportion, showing no separation between DS persons and healthy adults. Blue, DS persons; red, healthy adults [24].

**Figure 3:**
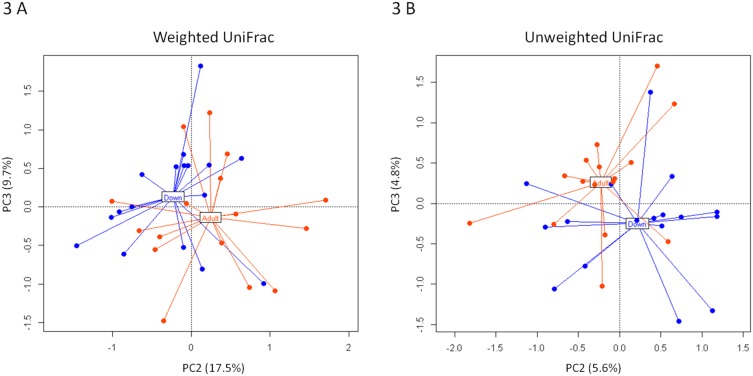
Weighted (A) and unweighted (B) UniFrac distance PCoA of the fecal microbiota of DS persons and healthy non-trisomic adults. Percentage of variance in the dataset shown by the second and third principal component (PC) is reported. Blue, DS persons; red, healthy adults [24].

According to our data, DS persons and healthy adults showed comparable levels of GM diversity and an overall similarity in the composition of the GM community (Figure 2). The visualization of community structures using PCoA of weighted UniFrac distances showed only a weak segregation between the two groups (*P = *0.28), confirming the overall similarity of the two GM ecosystems (Figure 3A). However, the PCoA of unweighted UniFrac distances tended to separate the two subject groups (Figure 3B) (*P = *0.09). This suggests that GM differences between DS persons and healthy controls principally involved subdominant GM components. Indeed, within the dominant GM families no significant differences in rel. ab. between the two groups of subjects were detected. Differently, DS persons were characterized by a higher abundance in several subdominant GM genera, such as *Parasporobacterium* (*P = *0.036) and *Sutterella* (*P = *0.012), as well as by a reduction in the rel. ab. of the family *Veillonellaceae* (*P = *0.005).

In order to explore possible alterations of the GM of DS persons matching an aged-type ecosystem, we performed a meta-analysis of the GM structure also including 5 healthy Italian elderly and 3 centenarians from Rampelli *et al.* [25]. The PCoA of the Euclidean distances of the genus-level GM composition in DS persons, healthy adults, elderly (mean age 66.4 years) and centenarians (mean age 100.7 years) is provided as Figure S3. Data shows that DS persons and heathy adults segregate together, suggesting that the gut microbial ecosystem of DS persons does not show the typical deviations of an aged-type gut microbial community.

### Correlation between ABC score and signature genera of DS microbiome

Since microbiota dysbioses have been recently associated with certain neuro-behavioral disorders [33], here we sought Kendall correlations between ABC or VABS total score in DS persons and the three GM groups characterizing DS microbiome: *Parasporobacterium*, *Sutterella* and *Veillonellaceae.* The values of ABC and VABS scores for the enrolled DS persons are reported in Table S3. While no significant correlations were found between signature DS microorganisms and VABS score, which is indicative of the adaptive behavior of DS persons, *Sutterella* was positively correlated with the ABC total score (tau = 0.40, *P*<0.05). On the other hand, no correlation was found between ABC and *Parasporobacterium* as well as *Veillonellaceae*.

## Discussion

Hypothesizing that the layout of the GM in DS persons could be of some relevance for their health, here we investigated the GM structure of 17 DS persons. In order to highlight signatures of an aged-type microbiota in DS, the GM profile of DS persons was compared with that of age-matched healthy non-trisomic Italian adults, and healthy Italian elderly and centenarians. According to our finding, DS persons and healthy adults possessed a similar complement of dominant groups in the GM, showing an overall healthy-like microbiota profile without any signs of premature microbiota aging [34].

The GM of DS persons was found to be well forged to provide the host with short-chain fatty acids (SCFA), butyrate, propionate and acetate, from fermentation of indigestible polysaccharides in the gut. Indeed, DS microbiota was largely dominated by the butyrate producers *Ruminococcaceae* (*Clostridium* cluster IV) and *Lachnospiraceae* (*Clostridium* cluster XIVa) [35], propionate-producing Bacteroidetes [36], and the acetate producer *Bifidobacterium* [37], [38]. SCFA are pivotal metabolites in the microbiota-host immunological cross-talk, and their specific role as immune system modulators has been recently proved. In particular, butyrate is crucial to promote the extrathymic differentiation of regulatory T cells (Treg), whereas acetate is functional to drive their accumulation in the colon. Propionate exerts a dual function, capable to boost peripheral Treg generation and subsequent homing in the colon [39]. Thus, it is tempting to speculate that - by providing the host with SCFA from dietary polysaccharides - the gut microbiome of DS persons could be strategic to counteract the pro-inflammatory immune defects driving to immunosenescence [40]. This could explain why signs of premature immunosenescence found at the level of lymphocyte subpopulations (decreased B cells and T cells, in particular CD8+ T cells, and increased NK cells) are not apparently paralleled by the other major event of immunosenescence, *i.e.* the increased level of pro-inflammatory molecules. In any case, due to the limited number of DS persons enrolled in the present study, these assumptions need to be taken with extreme caution. Moreover, it is to note that the relatively young age of the subjects studied here (max 35 years) does not allow us to exclude that later on this event takes place anyway. Thus, further studies enrolling more DS persons and including older subjects are needed to prove these hypotheses, however, should it turn to be confirmed, it will make GM a strategic factor to counteract the genetically determined acceleration of the process of immunosenescence of DS persons. Within this context, it will become of extreme relevance to control diet in DS persons, supporting the maintenance of their healthy profile of the dominant GM. For instance, the consumption of a fiber-containing diet, which favors immunomodulating GM actors as *Clostridium* clusters IV and XIVa [41], should be encouraged.

While the general GM structure of DS persons was comparable to the one of healthy subjects, we observed significant differences among the subdominant groups. In particular, according to our findings, DS persons were enriched in *Parasporobacterium* and *Sutterella*, and reduced in *Veillonellaceae*. The increase of *Sutterella* and the reduction of *Veillonellaceae* have been described in autistic children with gastrointestinal symptoms [22], [23], [42]. In our study, the abundance of *Sutterella* in the GM was positively correlated with the ABC total score in DS persons, suggesting a possible link between this microorganism and ASD in DS [21], [43]. As a Gram-negative opportunistic pathogen, *Sutterella* has been proposed to aggravate ASD through LPS-induced inflammation in the brain [44]. Conversely, the connection between *Veillonellaceae* and autistic symptoms was apparently less obvious. However, according to Kang *et al.* [42], the reduction of this group in the gut was more closely linked to the presence of autistic symptoms, rather than to the severity of gastrointestinal symptoms or the specific dietary regimen.

The correlation between the relative abundance of *Sutterella* in the GM and ABC total score in DS persons further strengthens the hypothesis in favor of a possible role for this gut microorganism in maladaptive behavior, opening the way to further investigation on the role of GM dysbioses in adults with ASD.

## Supporting Information

Figure S1 **Alpha-diversity rarefaction curves for the 16S rRNA V4 region pyrosequencing reads.** The OTU table was rarefied up to 2,500 reads per sample and analyzed using various diversity metrics for each enrolled DS person. Metrics used were the Chao1 index of microbial richness, observed species, Faith’s phylogenetic diversity index (PD whole tree), and the Shannon index of biodiversity. The individual rarefaction curves are color-coded according to the list of DS persons on the right.
(TIF)

Figure S2 **PCoA of the unweighted Unifrac distances of the fecal microbiota of DS persons grouped according to body mass index (BMI).** The BMI cutoff point of ≥25 kg/m^2^ for overweight was used [31]. Green, BMI <25 kg/m^2^; yellow, BMI ≥25 kg/m^2^.
(TIF)

Figure S3 **PCoA of the fecal microbiota in DS persons, healthy adults, elderly and centenarians.** PCoA was based on the Euclidean distances of the relative abundance of gut microbiota genera in DS persons from the present study (green), healthy adults from Schnorr *et al.* [24] (blue), healthy elderly (red) and centenarians from Rampelli *et al.* [25] (orange).
(TIF)

Table S1 Demographic and clinical features of the enrolled Down Syndrome persons.
(DOCX)

Table S2 Percent contribution of food categories to the diet of Down Syndrome persons.
(DOCX)

Table S3 Level of cognitive impairment, Aberrant Behavior Checklist (ABC) and Vineland Adaptive Behavior Scale (VABS) scores in the enrolled Down Syndrome persons.
(DOCX)
