# Supplementary figures and images for: Gut Microbiome in Down Syndrome
Source: PLoS One. 2014 Nov 11;9(11):e112023. doi: 10.1371/journal.pone.0112023 (PMC4227691; doi:10.1371/journal.pone.0112023)

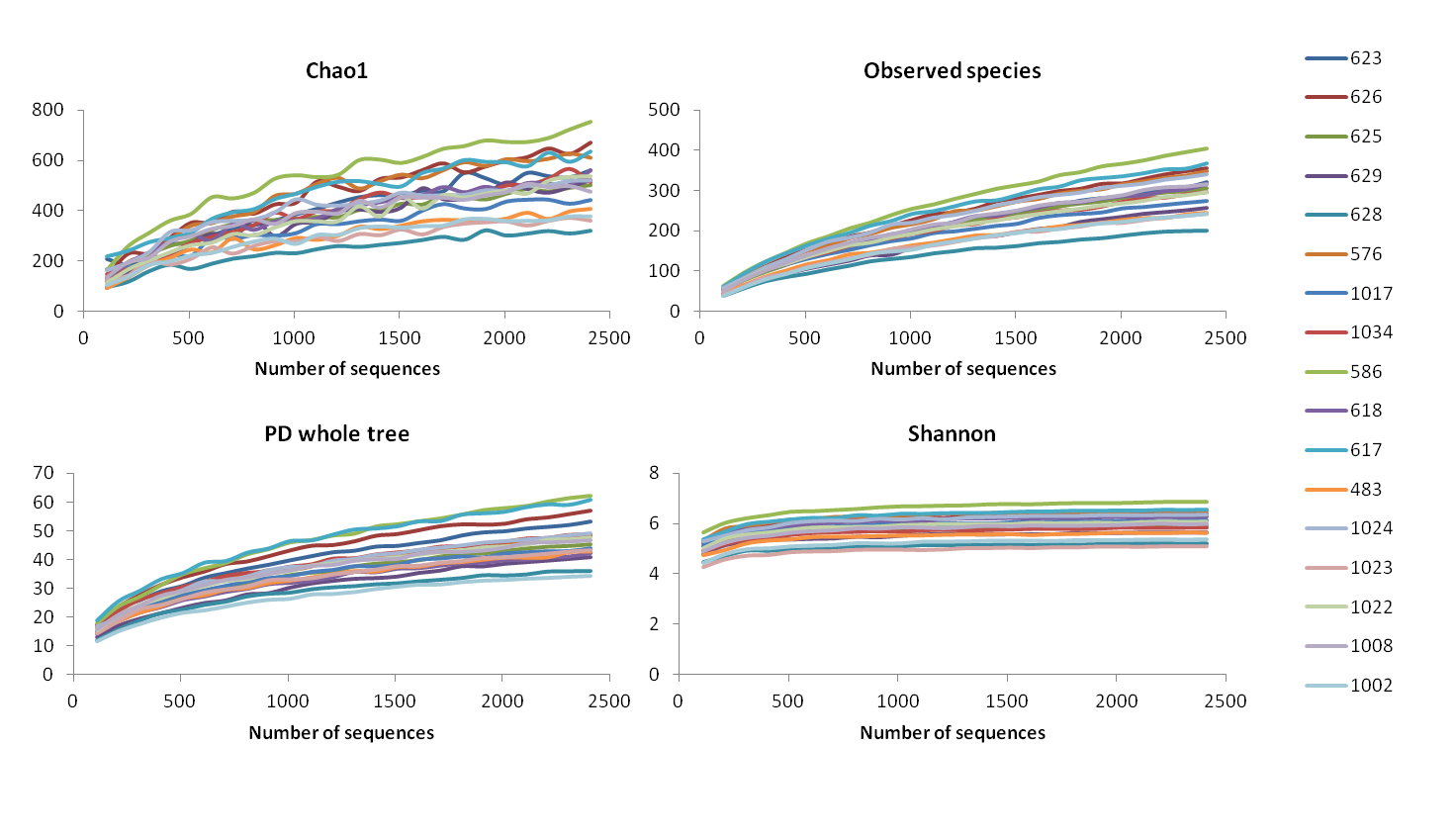

Supplement: Figure S1 — Alpha-diversity rarefaction curves for the 16S rRNA V4 region pyrosequencing reads. The OTU table was rarefied up to 2,500 reads per sample and analyzed using various diversity metrics for each enrolled DS person. Metrics used were the Chao1 index of microbial richness, observed species, Faith’s phylogenetic diversity index (PD whole tree), and the Shannon index of biodiversity. The individual rarefaction curves are color-coded according to the list of DS persons on the right. (TIF) [file pone.0112023.s001.tif]

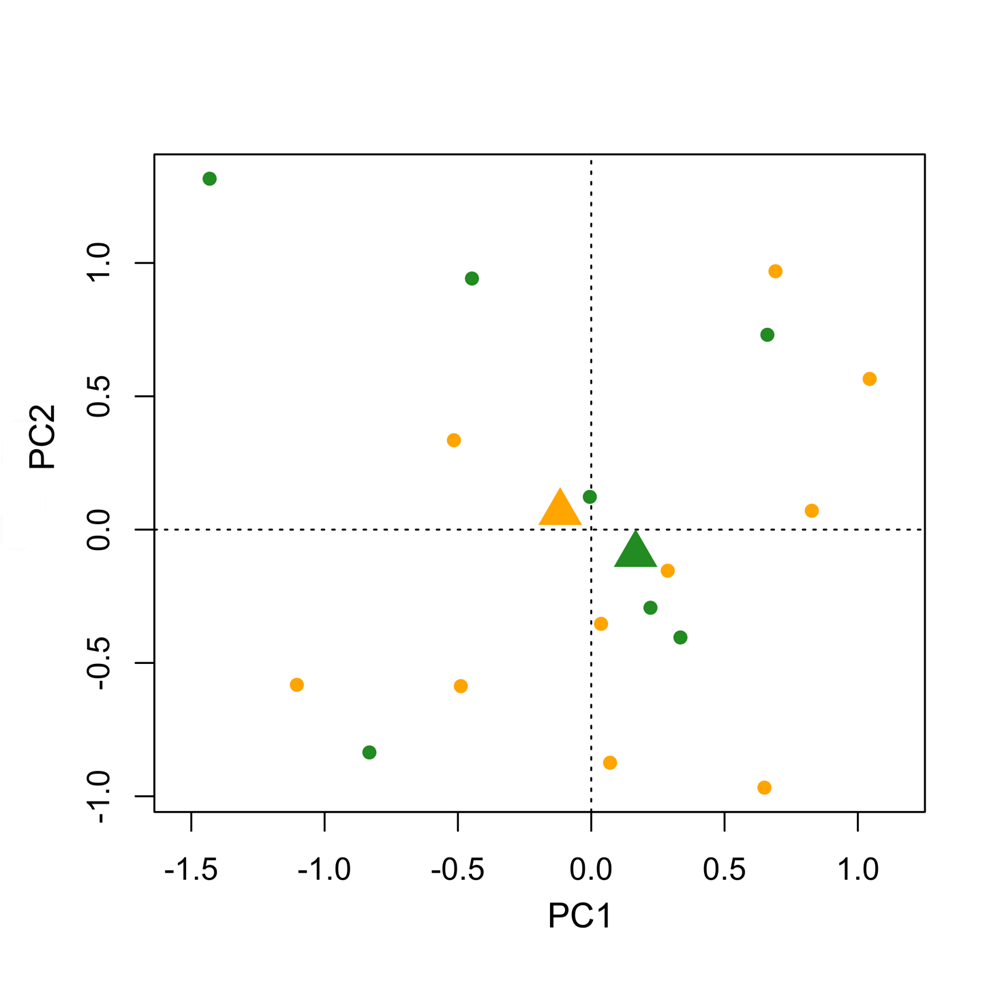

Supplement: Figure S2 — PCoA of the unweighted Unifrac distances of the fecal microbiota of DS persons grouped according to body mass index (BMI). The BMI cutoff point of ≥25 kg/m2 for overweight was used [31]. Green, BMI <25 kg/m2; yellow, BMI ≥25 kg/m2. (TIF) [file pone.0112023.s002.tif]

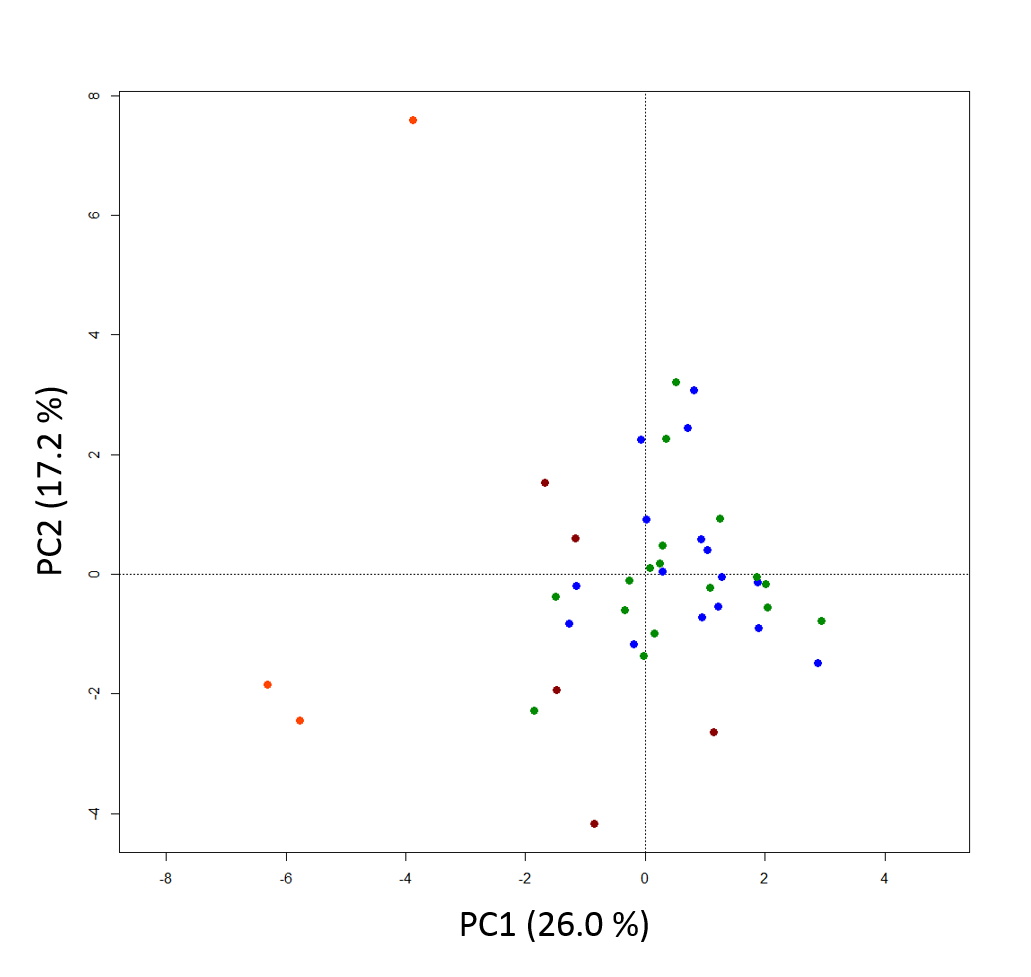

Supplement: Figure S3 — PCoA of the fecal microbiota in DS persons, healthy adults, elderly and centenarians. PCoA was based on the Euclidean distances of the relative abundance of gut microbiota genera in DS persons from the present study (green), healthy adults from Schnorr et al. [24] (blue), healthy elderly (red) and centenarians from Rampelli et al. [25] (orange). (TIF) [file pone.0112023.s003.tif]
