# Supplementary material for: Gut Microbiome in Down Syndrome
Source: PLoS One. 2014 Nov 11;9(11):e112023. doi: 10.1371/journal.pone.0112023 (PMC4227691; doi:10.1371/journal.pone.0112023)
Supplement: Table S1 — Demographic and clinical features of the enrolled Down Syndrome persons. (DOCX) [file pone.0112023.s004.docx]

**Table S1.** Demographic and clinical features of the enrolled Down Syndrome persons.

| **SampleID** | **Age**  **(years)** | **Gender** | **Height**  **(cm)** | **Weight**  **(kg)** | **Belly**  **(cm)** | **BMI** | **Weight classification** | **Dietary habits**  **(food intake)** | **GI problem** | **Karyotype** | **LT4** |
| --- | --- | --- | --- | --- | --- | --- | --- | --- | --- | --- | --- |
| 1008 | 30 | M | 153 | 64.5 | 89 | 27.55 | overweight | excessive | none | trisomy 21 | y |
| 1024 | 28 | M | 154 | 62 | 81 | 26.14 | overweight | excessive | none | trisomy 21 | y |
| 1034 | 24 | F | 138 | 45 | 67.5 | 23.63 | normal weight | normal | constipation | trisomy 21 | y |
| 1002 | 24 | M | 159 | 76 | 93 | 30.06 | obesity | excessive | milk intolerance | trisomy 21 | n |
| 1022 | 24 | M | 149 | 50.7 | 76 | 22.84 | normal weight | normal | none | trisomy 21 | n |
| 1023 | 30 | F | 140 | 50 | 72 | 25.51 | overweight | excessive | none | trisomy 21 | n |
| 1017 | 30 | F | 140 | 48.5 | 70 | 24.74 | normal weight | normal | none | trysomy 21 | n |
| 483 | 35 | F | 142 | 66 | 89 | 32.73 | obesity | normal | milk intolerance | mosaic | y |
| 617 | 27 | M | 152.5 | 72 | 99 | 30.96 | obesity | excessive | constipation | trisomy 21 | y |
| 629 | 19 | F | 141 | 64 | 85 | 32.19 | obesity | normal | none | trisomy 21 | y |
| 576 | 34 | F | 149 | 50 | 76 | 22.52 | normal weight | normal | constipation, diarrhoea, gastritis | translocation | n |
| 618 | 26 | F | 142 | 48 | 76 | 23.8 | normal weight | normal | diarrhoea | trisomy 21 | n |
| 623 | 26 | M | 148 | 62 | 89 | 28.31 | overweight | normal | none | trisomy 21 | n |
| 625 | 22 | M | 152 | 58 | 77 | 25.1 | overweight | normal | none | trisomy 21 | n |
| 626 | 26 | F | 150 | 84.5 | 102 | 37.56 | obesity | normal | constipation | trisomy 21 | y |
| 586 | 30 | F | 142 | 46 | 80 | 22.81 | normal weight | normal | constipation, GER | missing data | y |
| 628 | 19 | F | 132.5 | 41 | 66.5 | 23.35 | normal weight | normal | constipation, GER | mosaic | n |

BMI, body mass index (kg/m^2^); GI, gastrointestinal; GER, gastroesophageal reflux; LT4, levothyroxine; y, yes; n, no.
