# Supplementary material for: Gut Microbiome in Down Syndrome
Source: PLoS One. 2014 Nov 11;9(11):e112023. doi: 10.1371/journal.pone.0112023 (PMC4227691; doi:10.1371/journal.pone.0112023)
Supplement: Table S2 — Percent contribution of food categories to the diet of Down Syndrome persons. (DOCX) [file pone.0112023.s005.docx]

**Table S2.** Percent contribution of food categories to the diet of Down Syndrome persons.

| **SampleID** | **Bread, cereal, pasta, grain** | **Legumes, rice, potatoes** | **Meats, egg** | **Dairy products** | **Fruit** | **Vegetables** | **Desserts** |
| --- | --- | --- | --- | --- | --- | --- | --- |
| 1008 | 29 | 5 | 1 | 12 | 19 | 19 | 13 |
| 1024 | 38 | 3 | 10 | 13 | 3 | 21 | 11 |
| 1034 | 30 | 5 | 4 | 16 | 20 | 20 | 5 |
| 1002 | 47 | 2 | 16 | 13 | 8 | 3 | 13 |
| 1022 | 25 | 3 | 6 | 22 | 22 | 22 | 1 |
| 1023 | 36 | 4 | 12 | 20 | 3 | 24 | 2 |
| 1017 | 33 | 3 | 9 | 15 | 18 | 18 | 5 |
| 483 | 28 | 5 | 18 | 27 | 4 | 14 | 5 |
| 617 | 37 | 4 | 7 | 15 | 24 | 8 | 4 |
| 629 | 29 | 2 | 22 | 13 | 14 | 14 | 5 |
| 576 | 29 | 1 | 6 | 18 | 21 | 21 | 4 |
| 618 | 32 | 1 | 17 | 14 | 22 | 11 | 3 |
| 623 | 15 | 2 | 19 | 31 | 15 | 15 | 2 |
| 625 | 22 | 4 | 25 | 14 | 11 | 22 | 3 |
| 626 | 32 | 1 | 23 | 21 | 10 | 10 | 1 |
| 586 | 28 | 2 | 11 | 21 | 19 | 19 | 1 |
| 628 | 22 | 6 | 17 | 18 | 11 | 22 | 6 |
| **MEAN** | **30** | **3** | **13** | **18** | **14** | **17** | **5** |
