# Supplementary material for: Gut Microbiome in Down Syndrome
Source: PLoS One. 2014 Nov 11;9(11):e112023. doi: 10.1371/journal.pone.0112023 (PMC4227691; doi:10.1371/journal.pone.0112023)
Supplement: Table S3 — Level of cognitive impairment, Aberrant Behavior Checklist (ABC) and Vineland Adaptive Behavior Scale (VABS) scores in the enrolled Down Syndrome persons. (DOCX) [file pone.0112023.s006.docx]

**Table S3.** Level of cognitive impairment, Aberrant Behavior Checklist (ABC) and Vineland Adaptive Behavior Scale (VABS) scores in the enrolled Down Syndrome persons.

| **SampleID** | **Cognitive impairment** | **ABC**  **irritability** | **ABC**  **lethargy** | **ABC**  **stereotypy** | **ABC**  **hyperactivity** | **ABC**  **inappropriate speech** | **ABC**  **TOTAL SCORE** | **VABS communication** | **VABS**  **daily living skills** | **VABS socialization** | **VABS**  **motor skills** | **VABS**  **TOTAL SCORE** |
| --- | --- | --- | --- | --- | --- | --- | --- | --- | --- | --- | --- | --- |
| 1008 | mild | 0 | 0 | 4 | 3 | 5 | 12 | 125 | 125 | 134 | 112 | 128 |
| 1024 | moderate | 7 | 6 | 10 | 8 | 10 | 41 | 86 | 104 | 97 | 113 | 101 |
| 1034 | mild | 0 | 0 | 0 | 0 | 4 | 4 | 120 | 125 | 132 | 115 | 126 |
| 1002 | severe | 0 | 1 | 2 | 2 | 2 | 7 | 105 | 108 | 120 | 112 | 113 |
| 1022 | mild | 4 | 0 | 3 | 0 | 5 | 12 | 121 | 123 | 127 | 115 | 125 |
| 1023 | moderate | 2 | 10 | 2 | 7 | 4 | 25 | 112 | 122 | 113 | 105 | 115 |
| 1017 | mild | 0 | 1 | 2 | 0 | 4 | 7 | 107 | 115 | 107 | 106 | 111 |
| 483 | mild | 1 | 7 | 2 | 0 | 2 | 12 | 125 | 137 | 142 | 116 | 134 |
| 617 | mild | 2 | 8 | 2 | 1 | 4 | 17 | 121 | 128 | 113 | 115 | 122 |
| 629 | severe | 1 | 2 | 0 | 4 | 0 | 7 | 114 | 120 | 128 | 113 | 122 |
| 576 | mild | 0 | 0 | 0 | 0 | 0 | 0 | 126 | 139 | 141 | 118 | 135 |
| 618 | mild | 9 | 2 | 1 | 1 | 2 | 15 | 119 | 127 | 129 | 115 | 126 |
| 623 | mild | 1 | 2 | 0 | 0 | 5 | 8 | 115 | 114 | 126 | 109 | 119 |
| 625 | moderate | 3 | 7 | 0 | 12 | 3 | 25 | 94 | 115 | 112 | 117 | 111 |
| 626 | mild | 4 | 1 | 0 | 0 | 3 | 8 | 126 | 136 | 146 | 118 | 136 |
| 586 | severe | 0 | 2 | 6 | 10 | 3 | 21 | 102 | 98 | 96 | 114 | 104 |
| 628 | moderate | 1 | 11 | 0 | 1 | 5 | 18 | 85 | 116 | 106 | 112 | 106 |
